# Positive Adult Education, Learned Helplessness and the Pygmalion Effect

**DOI:** 10.3390/ijerph19020778

**Published:** 2022-01-11

**Authors:** David Cobos-Sanchiz, Manuel-Jesús Perea-Rodriguez, Juan-Agustín Morón-Marchena, María-Carmen Muñoz-Díaz

**Affiliations:** 1 Department of Education and Social Psychology, Universidad Pablo de Olavide, 41013 Seville, Spain; dcobos@upo.es (D.C.-S.); jamormar1@upo.es (J.-A.M.-M.); mcmundia@upo.es (M.-C.M.-D.); 2 Department Lifelong and Adult Education, Universidad Popular Dos Hermanas, 41700 Seville, Spain

**Keywords:** positive education, Pygmalion effect, learned helplessness, lifelong education, adult education

## Abstract

Positive education is seen as a transformative methodological approach capable of improving the act of teaching and learning and, above all, essential for the development of students’ personal skills and competences. However, few studies have been carried out on this topic in the field of adult and continuing education; instead, they have been published mainly in the field of formal education and at school age. This study works with a sample of 399 people over 16 years of age and students of the Universidad Popular de Dos Hermanas in order to show the relationship between the Pygmalion effect and learned helplessness in the process of acquiring knowledge in adulthood. In this way, three tools were used: one questionnaire that showed the teachers’ perceptions of the students’ qualities and behaviour and two that provided information on self-concept, self-esteem, personal and social skills and other variables directly related to emotional intelligence and positive education. It shows how exposure to negative operational constraints hinders the psychosocial and socio-educational development of learners in all possible ways, while, on the other hand, it indicates the importance of positive education to compensate for this phenomenon by enhancing the development and growth of those who study and participate in non-formal education through positive reinforcement. Likewise, the factorial interrelation of both positive and negative conditioning factors and their incidence on learning is shown; the importance of neutralising the negative components and strengthening the positive reinforcement and the role played by the community and education professionals as catalysts and behavioural modulators at any stage of learning and age group for the achievement of the objectives of the student and of education itself in a broad sense.

## 1. Introduction

The aim of this study is to provide keys for the improvement of the teaching–learning process, both in terms of the competences included in each of the modules and in terms of personal and social skills, based on two basic ideas: learned helplessness and the Pygmalion effect. Both are juxtaposed situations that interfere in the results of the learner and from which education professionals can establish mechanisms for educational improvement.

With this, the aim is to show the value of positive education as a guiding and transforming element, not only in the teaching–learning process, but also in the quality of personal and community life, as well as in social cohesion. That is, taking into account the evidence of how emotional intelligence and personal and social skills training in turn have a positive impact on the social climate, the classroom, interpersonal relationships, inclusion, motivation, prevention of disruptive behaviour and attitudes, etc. [1,2,3,4,5]. In addition, following the postulates of operant conditioning which holds that behaviour can be modified by reinforcement determining their effects on learning and response behaviour [6]; we find the basis for a methodological model that allows the modulation of those elements involved in the cognitive and academic processes that are affected by internal [7] and external variables [8,9] favouring or neutralising personal growth. This shows the symbiotic relationship between emotions and the teaching–learning process [10].

In the 1960s, [11] Seligman coined the term learned helplessness to show how the use of negative reinforcement was key to overriding the capacity for reaction and behavioural shaping in animals, which was later tested in humans [12,13]. In this way, it was observed that those who had been exposed to aversive situations tended towards avoidance and ultimately to the belief that no matter how they operated, no positive outcome would be obtained. Thus, it is a model of uncontrollable stress that produces emotional distress [14,15], and as a coping deficit with characteristics of depression [14,16] in the face of aversive but avoidable situations [17].

Its conceptualisation has evolved over the years, resulting in several theses over time. In its beginnings, this theory was used to expose both animal behaviour [18,19,20,21,22,23,24,25] and human behaviour in learning and coping processes, being part of an experiment in behavioural situations, passing to the present day as a model that generates many other theories in areas such as education or health and related to psychological problems in human beings.

For [26] Peterson, Maier and Seligman, the theory of learned helplessness constitutes three essential elements: contingency, cognition and behaviour.

- Contingency: is the objective correspondence between an individual’s action and the outcomes derived from experience. Contingency can be described in two constructs: controllability and lack of control.
- Cognition: is understood as the way in which a person perceives, explains and extrapolates contingency [27]. The process of cognition is composed of several stages in which the person first understands the contingency.
- Behaviour: the observable consequences of non-contingency and the individual’s cognitions about it [27].

Considering this, by definition, learned helplessness studies measure the passivity of an individual’s activity in a situation different from the one in which uncontrollability was first encountered.

On the other hand, this model argues that there are other consequences of a person’s expectations of future helplessness: cognitive retardation, low self-esteem, loss of aggression, immunological changes and physical illness [26]. 

Its opposite is the Pygmalion effect, based on the Pygmalion or fulfilled prophecy myth, taken by cognitivism to explore how positive reinforcement and realistically displayed expectations of success influence the learner to achieve success, thereby improving outcomes. From the above, we can see the importance of any type of conditioning and emotional learning for the development of the person, taking into account the transversal axis: motivation–cognition–emotion, which in turn connects with brain dysfunctions caused by the stimulus.

This approach received impetus from the experiment conducted by [28] Robert Rosenthal and Lenore Jacobson, where the expectation of an experimenter strongly influenced the way subjects behaved in different situations. 

For [29] Rosenthal, there are three factors that influence the Pygmalion effect:

- Climate: a warmer climate is created for those students from whom more is expected.
- Input: teachers teach more things to students from whom they expect more.
- Feed-back: the more is expected from students, the more praise and positive reinforcement they receive: there is a direct relationship between more praise and positive reinforcement.

Sánchez Hernández and López Fernández [30] define it as follows: the Pygmalion effect requires three aspects: firmly believing in a fact, having the expectation that it will be fulfilled and accompanying it with messages that encourage its achievement. 

We know the Pygmalion effect as the prophecy of self-realisation, which can have both an external and an internal origin. Success, with respect to this subject, lies in the capacity that both subjects, as well as the people around them, have to be able to create positive expectations, firmly believing in them and transmitting them with the same intensity, turning them into a powerful stimulus. 

Therefore, and on the basis of positive education in which the present analysis is embedded, we show how to approach the teaching–learning act with adults, taking into account the different conditioning models that the learner brings internalised, as well as the elements contemplated in the model of Goleman, Mayer et al. [31] such as: emotional intelligence, self-esteem, resilience, self-efficacy and empathic attitudes; and on the other hand, symptoms such as anxiety and stress [32,33,34,35,36]. At the same time, it is evident how the power of contextual elements [37,38], reinforcement [39], motivation and expectations about reinforcement [40,41,42,43,44] will significantly influence meaningful learning [45], especially positive feedback [46].

Education based on emotional intelligence is necessary for students to be able to positively use and control their emotions, with positive coping strategies for stressful situations [47] and triggers [48], improving their psychological well-being [49]; which in turn promotes a feeling of belonging to a group [50,51,52], greater personal, organisational and social success [53,54], with a strong positive impact on the community [55].

The study was carried out at the Universidad Popular de Dos Hermanas, an institution created in the city in 1992 and whose area of work is adult education on the basis of “Education for all” and “throughout life”; working in turn in different areas of non-formal education: socio-cultural animation and personal and community development [56]. It should be noted that its general objectives include, among others: facilitating access to education for those who for various reasons have been left out of the education system; improving the socio-labour profile of those who attend; promoting education in democratic values; developing transversal strategies for inclusive education, attending to diversity and from a gender perspective; enhancing personal and social skills through learning; and promoting the empowerment and awareness of the individual to improve the quality of life at all stages of adult life and old age [57].

## 2. Materials and Methods

In order to gather the relevant information on the degree of learned helplessness and the Pygmalion effect, this research provides clues for the improvement of the teaching–learning process of the knowledge in which the adults in this centre are enrolled and of personal and social skills, with three specific objectives:

- To expose whether there are deficiencies related to social skills and self-esteem and, therefore, learned helplessness.
- To show whether behaviour correlates with learned helplessness or whether it can be modified to improve learning and reduce psychosocial risks.
- To show the keys of the Pygmalion effect to compensate the negative effects of learned helplessness as a scaffolding for the improvement and development of personal and social skills and empowerment.

Regarding the methodology established to carry out the research, in order to extract all the necessary information related to such broad study questions, we agreed on a combination of methods. Thus, the methodology is mixed qualitative and quantitative [58]. 

The research is made up of three compilations, on the one hand, a specific questionnaire for teachers (28 persons), where the behaviour of students was collected, with a total of 15 items, gathering the information related to the 399 persons that made up the sample. This was completed by all the teaching staff at the centre, in order to ascertain the influence of these variables in an objective and triangulated manner. 

On the other hand, a questionnaire on self-esteem and social skills, consisting of 15 items each, was reformulated for the students of the Universidad Popular de Dos Hermanas. The questionnaire is a reformulation and adaptation of the questionnaire for women by Castillo, S., et al. [59], with the addition of other questions divided into appendices. It has tried to combine personal skills and experiences, while questionnaires on self-esteem, social skills, social participation and interests and habits have been introduced. In this way, the same tool could be used to assess their concerns, well-being and health, aptitudes, shortcomings and opportunities.

The questionnaire explained the objective of the research and specified that the answers would be anonymous. Of the 1356 people enrolled in the 2020–2021 academic year, a total of 399 were included in the sample, in accordance with the representative characteristics of this population, who successfully completed the questionnaire, the sample having a confidence level of 95% in relation to the total population. Thus, the sample was made up of 399 people over 16 years of age, proportionally representing the institution’s target public, following quotas of gender, age, experience at the university, areas of knowledge and origin of the students, in order to maintain coherence with the reality of the centre itself.

For data processing and analysis, all the information in the database was entered into the statistical programme SPSS, version 24 [60].

The validity of the questionnaires is supported by previous research by their creators; likewise, as it is a reformulation, we evaluated the coherence and internal consistency of the tool. On the other hand, Cronbach’s alpha coefficient [61] was used to study the reliability of the tool, with values of 0.941, 0.831 and 0.805, respectively. Following the general recommendation of George and Mallery [62], based on the scores obtained in each of the questionnaires, according to Cronbach’s alpha, it can be concluded that the instrument used was very suitable for the study, as the items measured the same construct and were highly correlated.

In this process of data collection, triangulation was fundamental in carrying out the research. Firstly, the data provided by the Universidad Popular itself in terms of statistics and reports were taken into account. Based on this, the identification and analysis of triggering situations was carried out, as well as the selection and adaptation of techniques, tools and instruments for the collection and elucidation of data.

The third component of the study was the unstructured interviews with teachers, which have been key throughout the analysis. With them it was possible to evidence and affirm ideas that had been revealed through the rest of the techniques.

We agreed on this methodological process as we considered it the most appropriate to obtain answers to the objectives set out. It was necessary to know the perception of the pupils themselves, as well as that of the teaching staff and the pooling of these objective results with the data obtained through the semi-structured interviews, in order to avoid any type of bias, and to be able to triangulate the different information to be dealt with.

## 3. Results

We are now in the central phase of the process, where, after using the tools mentioned above, the necessary information has been compiled. In order to elucidate the data in line with the proposed objectives, descriptive analyses were carried out to find out the socio-demographic characteristics, social skills, self-esteem and behaviour in the teaching–learning scenario. Within these descriptive analyses, the frequency and cumulative percentages were verified, as well as cross-checking tables with the chi-square test to clarify possible relationships between variables. The reliability of the internal consistency of the study factors was calculated by means of Cronbach’s alpha coefficient.

The sample consisted of 28% men and 72% women, with the widest age range being 45 to 54 years for women and 55 to 64 years for men. Of these, 36.1% were currently working and only 13.2% were retired or pensioners; 50.7% were unemployed. With regard to the level of studies, 20.1% had no qualifications, 19.5% had primary education, 28% had secondary education or BUP, 23% had studied vocational training and 10% had higher education.

**Figure 1:**
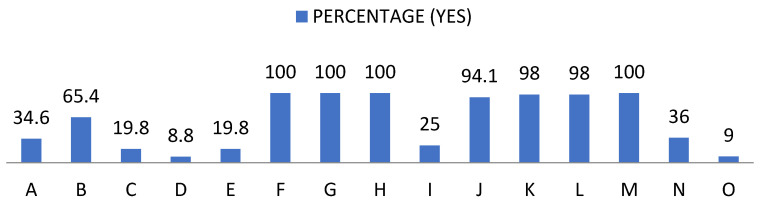
Percentage of “Yes” obtained from the social skills questionnaire. For the understanding of Figure 1 and Figure 2, the items observed in them correspond to: Items that are part of the social skills questionnaire. A: Possibilities of success. B: Personal limitations. C: Self-concept. D: Hetero concept. E: Skills. F: Interests. G: Social skills. H: Rights. I: Communication. J: Objectives. K. Extreme situations. L: Behaviour. M: Empathy. N: Reject requests. O: Express feelings.

**Figure 2:**
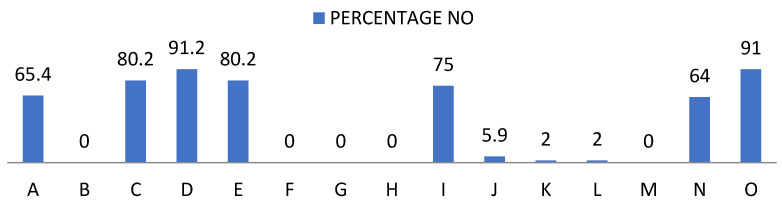
Percentage of “No” obtained from the social skills questionnaire.

The first set of data, relating to social skills, showed significant and revealing frequencies regarding the pupils’ self-perception, as shown below (Figure 1 and Figure 2):

- Of the pupils, 65.4% said they did not recognise their chances of success, compared to 100% who claimed to recognise their limitations.
- A proportion of 80.2% did not have a positive self-concept and said that they did not know how to identify their abilities and aptitudes. At the same time, 91.2% said they did not know how other people perceived them.
- One hundred percent showed willingness to make an effort to train their social skills, respect their rights and the rights of others, knew that they should behave differently depending on the situation and were able to put themselves in other people’s shoes.
- A proportion of 75% said they do not clearly communicate their wishes, opinions, feelings and goals; 94.1% said that they achieved their goals to the extent that they depend on them; 98% said that they tried to maintain or improve their relationship with others in extreme situations. A further 64% were not able to refuse requests and 91% did not express their feelings without difficulty.

**Figure 3:**
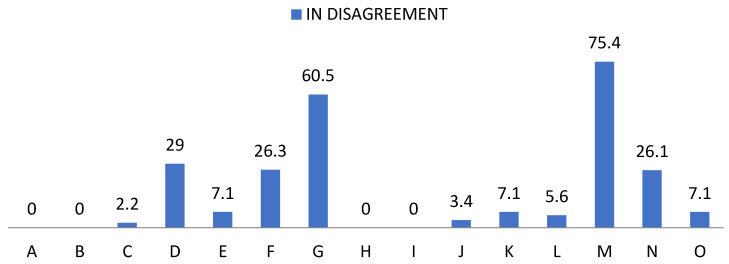
Percentage of “Disagree” from the self-esteem questionnaire. For the understanding of Figure 3, Figure 4 and Figure 5, the items observed in them correspond to: Items that are part of the self-esteem questionnaire. A: Appreciation. B: Failure. C: Qualities. D: Dignity. E: Pride. F: Positive attitude. G: Satisfaction. H: Value. I: Undervalue. J: Inequality. K: Change of attitude. L: Hide feelings. M: Discomfort. N: Fear of losing sympathy. O: Fear of rejection.

**Figure 4:**
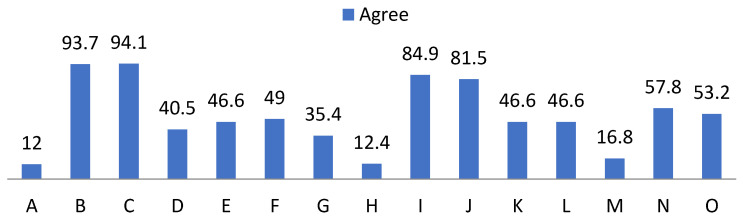
Percentage of “Agree” from the self-esteem questionnaire.

**Figure 5:**
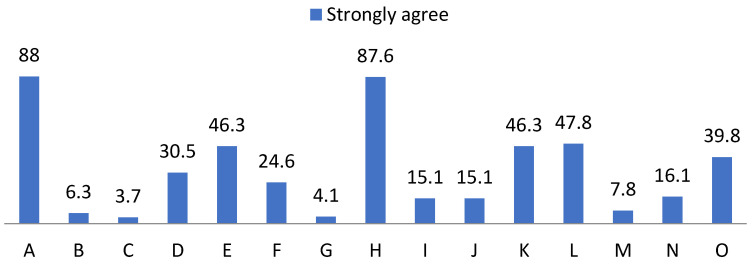
Percentage of “Strongly agree” from the self-esteem questionnaire.

With regard to the self-esteem questionnaire, high frequencies showing weakness and learned helplessness, as well as those that become basic pillars for positive reinforcement are noteworthy, as shown in Figure 3, Figure 4 and Figure 5.

**Figure 6:**
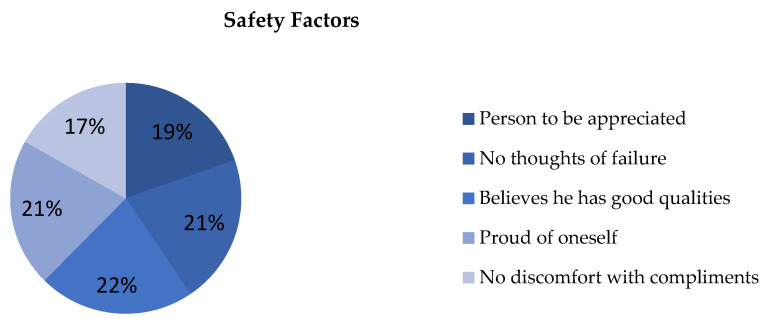
Percentage safety factors.

Taking into account the nature and impact of the variables, we segregated between risk factors and safety factors to summarize the influence of both groups in the teaching–learning process, as indicated in Figure 6.

In relation to risk factors, 30% did not feel that they were treated with the same dignity as the rest, 26.3% of the participants stated that they did not maintain a positive attitude towards themselves, while 60.5% did not feel satisfied with themselves. A proportion of 87.6% strongly agreed that they should be valued, in addition to the 12.4% who claimed to agree. On the other hand, 84.9% agreed that sometimes they felt that they were worthless and the remaining 15.1% strongly agreed.

Regarding the perception of their rights, 96.6% agreed and strongly agreed that they felt that they had fewer rights than other people.

Attending to assertiveness we found that:

- 92.9% found it difficult to ask another person to change her behavior if it was bothering them;
- 94.4% hid their feelings for fear of rejection;
- 83.9% hid ideas and opinions for fear of losing the sympathy of other people;
- 92.9% feared rejection reactions from people;
- 24.6% believed they received praise for pity or disability.

**Figure 7:**
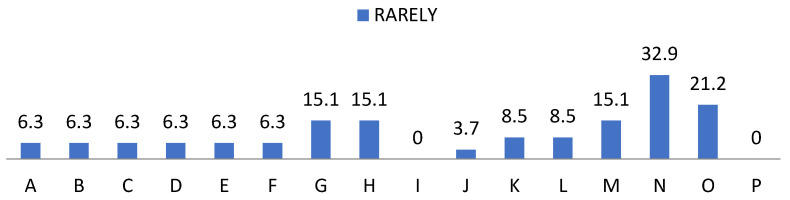
Percentage of “Rarely” from the teacher perception questionnaire. For the understanding of Figure 7, Figure 8, Figure 9 and Figure 10, the items observed in them correspond to: Items that are part of the teacher perception questionnaire. A: Available. B: You win. C: Attitude. D: Intent. E: Interest. F: Motivation. G: Compression. H: Expression. I: Aptitude. J: Behaviour. K: Inventiveness. L: Restlessness. M: Communication. N: Over traction. O: Participation. P: Learning.

**Figure 8:**
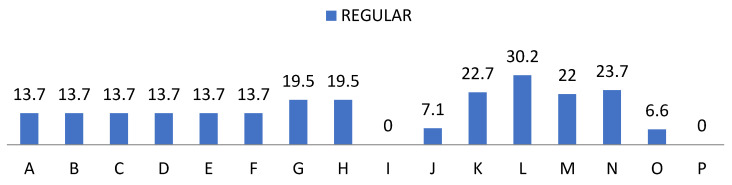
Percentage of “Regular” from the teacher perception questionnaire.

**Figure 9:**
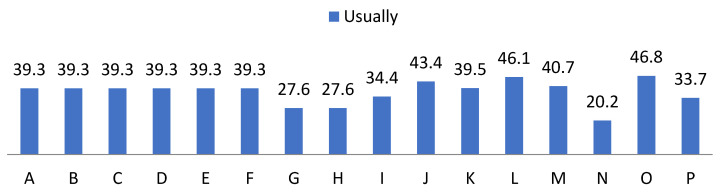
Percentage of “Usually” from the teacher perception questionnaire.

**Figure 10:**
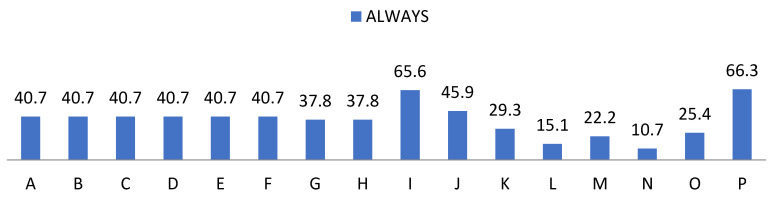
Percentage of “Always” from the teacher perception questionnaire.

On the other hand, we show the data related to the behaviour of the students provided by the teaching staff in Figure 7, Figure 8, Figure 9 and Figure 10. In the items “disposition”, “desire”, “attitude”, “intention”, “interest” and “motivation” we found the same frequencies obtaining 80% values of almost always and always. Regarding “understanding” and “expression” we observed a cumulative frequency between almost never and regularly of 34.6%; as well as 49% participation in the same scores. Regarding “aptitude” we found 34.45% with almost always and 65.6% always; and “behaviour” obtained 43.4% almost always and 45.9% always; showing the item “learning” more frequently, obtaining 33.7% almost always and 66.3% always.

## 4. Discussion

This study focuses on the results of the questionnaires, where a ranking of perceived problems was obtained and contrasted with the teachers’ assessment. Taking into account the data presented in the results, we postulate as main problems and/or needs, the factors that were shown as variables of learned helplessness (unknown personal and social skills, lack of self-regulation, self-esteem and self-concept, inequality and lack of empowerment) to which we add proposals of attention for their resolution such as training in personal and social skills, training and coaching for the promotion of empowerment, recognition of capacities and aptitudes, learning in equal opportunities. 

These problems are the key elements in order of impact in the frequency tables after data processing, factors that are interrelated by their nature and that are framed within the same phenomenon with different stages or branches. Therefore, and taking into account the amount of data processed (both qualitative and quantitative), for the general overview it should be noted that the lack of formal training, role appropriation, diminished personal and social skills, low self-concept, self-esteem and self-regulation and low level of assertiveness for change are factors to be improved in order to achieve optimal levels of health and growth. In this way it is shown that, despite high levels of learned helplessness, the teacher’s assessment of the items “willingness”, “desire”, “attitude”, “intention”, “interest”, “motivation”, “attitude” and “aptitude” are crucial, based on those factors of counter security, to ensure that 100% of the students achieve a positive value in learning. 

This research, through the literature and the data obtained, highlights the importance of motivation and the elements that comprise it that have been previously reflected: both personal and environmental, expectations, self-efficacy, results, environment and social relations [63,64,65,66]. 

We also found that refocusing based on interests and external reinforcement improves student motivation in all facets of their lives [65]. With regard to conditioning factors [67], we should focus on those that will promote extrinsic motivation in accordance with positive education. Thus, we find that motivational, emotional and social elements are directly related to academic performance [68,69].

On the one hand, it is shown that the maintenance of a positive self-concept [70,71,72] and self-esteem [73,74,75] are vital because of their direct impact on motivation. 

In other words, the positive relationship between self-concept [76,77,78] and academic performance, as well as between self-esteem and performance [74,79,80], is ratified.

On the other hand, with regard to the emotional factors [81,82,83] understood as emotional intelligence, emotional competence and emotional well-being; added to the social characteristic of the human being and the act of teaching–learning itself, it highlights the need to maintain a trained personal and social interaction where the control of the internal locus allows the self-control of emotions and feelings [84,85]. The training and improvement of social intelligence, social competence and social skills are also necessary [86,87,88,89]. Similarly, there is a directly proportional relationship between academic performance and emotional intelligence [90,91,92].

As for the limitations of the research, on the one hand, the size of the sample, despite being representative and reliable, cannot be transferred to the bulk of the population as it represents the universe of students at the Universidad Popular. On the other hand, we cannot know the aetiology of the factors triggering learned helplessness in each of the cases in detail, but only verify that such dysfunction exists. Similarly, the longitudinal effect has to be taken into account, i.e., given the time of the research and the specificity of the analysis, we cannot show in this article the comparative pretest and post-test or control groups showing how the Pygmalion effect may stimulate change from baseline to completion.

However, these difficulties and limitations encountered are the threshold for future research. In such a way, the research was intended to find out in depth what the triggers of learned helplessness in adults are, and to know systematically how positive education affects behavioural modification, taking into account other variables.

## 5. Conclusions

Both the literature and the data extracted through the present research assert the importance of the impact of continued exposure to factors that inhibit intrinsic motivation. In the same way, we show how learned helplessness can be acquired independently of the personal and psychosocial profile, so that life history and the situational elements that affect personality development are key to growth and development in any environment. Therefore, it is shown that in the same way that external motivation influences internal motivation in a negative way, hindering and/or limiting development, the Pygmalion effect, i.e., positive support, becomes an engine of change for restructuring and cognitive refocusing for the optimal acquisition of tools and skills for life.

It is noteworthy that a large number of people are able to recognise their limitations but not their possibilities for success and claim not to have a positive self-concept and show low levels of assertiveness. On the other hand, they claim not to value themselves enough, which shows a diminished self-esteem and reveal in their answers how some social skills corresponding to participation and personal empowerment are dysfunctional. However, it is shown that the teachers’ perception of the students does not correspond to the initial idea that the students themselves have about themselves, and that through positive reinforcement, participation in educational actions and their learning achievements are fruitful. In other words, we find that it is common to show such learned helplessness in adulthood as a framework learned throughout life through conditioning and reinforcement.

Therefore, taking into account the association between emotions and attention, willingness to learn or behavioural self-regulation [93]; the relationship between personal well-being and emotional intelligence and academic performance [94], as well as the importance of satisfactory social relationships and social skills and performance [95,96], it is necessary to join efforts towards a new methodological approach based on positive education from childhood to neutralise these factors, thus achieving that in the evolutionary cycle, the adult person not only has access to new knowledge and learning, but that it is configured as a continuum of constant improvement and growth in all spheres of life, thus achieving a society whose emotional health is optimal and where the role of education is, in a broad sense, the perfectibility of the human being.

## Data Availability

Data available on request due to privacy and ethical restrictions. The primary data are contained within the article.
